# Prevalence of minimally invasive facial cosmetic surgery and its association with mental health among college students in Kuwait

**DOI:** 10.3389/fpubh.2025.1678308

**Published:** 2025-10-09

**Authors:** Eiman Alawadhi, Dina Ibrahim, Aishah Saadallah, Zainab Awada

**Affiliations:** ^1^Department of Epidemiology and Biostatistics, College of Public Health, Kuwait University, Al-Shadadiya, Kuwait; ^2^Faculty of Medicine, Department of Community Medicine and Behavioral Sciences, Kuwait University, Jabriya, Kuwait; ^3^Department of Population Health Sciences, University of Bristol, Bristol, United Kingdom

**Keywords:** minimally invasive facial cosmetic surgery, mental health, psychological health, college students, undergraduates, Kuwait University

## Abstract

**Background:**

Minimally Invasive Facial Cosmetic Surgery (MIFCS), which includes Botox and filler injections, is gaining popularity as a less invasive alternative to classic cosmetic procedures. Previous research has shown that psychological factors such as depression, anxiety, and self-esteem influence the decision to undergo MIFCS. This study aims to assess the relationship between mental health and undergoing MIFCS among undergraduate students attending Kuwait University (KU).

**Methods:**

We investigated the association between MIFCS and mental health variables in KU undergraduates. A cross-sectional design was employed to collect data from all 15 colleges at KU via an online questionnaire using convenience sampling. The study was approved by the Health Science Center (HSC) Center ethics committee at KU and Ministry of Health (MOH) in 9th of January 2025.

**Results:**

A total of 744 students participated, of whom 153 (20.6%) reported undergoing MIFCS. Across the entire sample, 64.4% reported having levels of depressive symptoms, 68.8% reported levels of anxiety symptoms, and 12.6% had low self-esteem. Factors associated with higher odds of MIFCS included being female (aOR 4.01, 95% CI 1.98–8.24), age over 20 years (aOR 4.20, 95% CI 2.47–7.16), being married (aOR 2.38, 95% CI 1.32–4.29), a history of physical disorders (aOR 1.77, 95% CI 1.11–1.66), a family history of mental disorders (aOR 1.87, 95% CI 1.06–3.31), and anxiety symptoms (aOR 2.27, 95% CI 1.42–3.64). In contrast, studying at the Health Sciences Center was associated with lower odds of MIFCS (aOR 0.44, 95% CI 0.25–0.76).

**Conclusion:**

The findings highlight the multifaceted nature of MIFCS and its substantial association with mental health issues among undergraduate students. Gender, age, marital status, and a personal or family history of physical and mental health disorders, particularly anxiety, are all key risk factors. Conversely, enrollment in medical disciplines was associated with lower odds of undergoing MIFCS. These findings highlight the importance of including mental health support and psychotherapy in clinical practices to offer holistic care for students’ well-being.

## 1 Introduction

Minimally invasive facial cosmetic surgery (MIFCS) procedures have gained increasing acceptance among healthy individuals without deformities due to their less invasive nature, reduced trauma, and fewer postoperative complications (1). There is a rising interest and trend among college students in undergoing Botox and filler injections (2, 3). Given this trend, our research primarily focused on undergraduate students.

Cosmetic procedures have been shown to influence mental health (4). Several studies consistently revealed an association between cognitive and psychological factors—including depression, anxiety, body image concerns, self-esteem, and self-efficacy —and a greater tendency for cosmetic surgery (4–9). Recent reviews have emphasized the importance of mental health screening in facial cosmetic surgery, highlighting its role in patient selection and outcomes (10). In addition to general psychological factors, body dysmorphic disorder (BDD) is a critical concern in the context of cosmetic surgery. Across studies from different countries, BDD affects approximately 2% of the general population but is reported in up to 15–20% of individuals seeking cosmetic procedures, making it one of the most important psychiatric conditions in this setting (10, 11). Patients with BDD are more likely to pursue cosmetic procedures but often experience poor postoperative satisfaction and deterioration in mental health. Furthermore, pre-existing mental health conditions, including depression, anxiety, and particularly BDD, can negatively influence postoperative outcomes, reduce patient satisfaction, and may even lead to worsening of psychological symptoms after cosmetic interventions (10).

Despite this, there is a noticeable gap in global literature concerning MIFCS and its association with psychological health. In Kuwait, a recent study among students at the College of Health Sciences at the Public Authority for Applied Education and Training (PAAET) revealed that 7.2% had undergone cosmetic surgery (12). However, the generalizability of these findings may be limited as the study focused exclusively on students with a scientific background. A broader study including students from diverse academic backgrounds is needed to better understand cosmetic surgery perceptions among college students in Kuwait. Furthermore, the existing literature on cosmetic surgery in Kuwait lacks studies connecting these procedures with mental health. Therefore, the current study aims to investigate the association between mental health and undergoing MIFCS among undergraduate students attending Kuwait University (KU).

## 2 Methods

### 2.1 Design, setting, and characteristics of participants

A cross-sectional study was designed to enroll participants from KU undergraduate colleges. KU includes 15 undergraduate colleges: College of Arts; Education; Law; Sharia and Islamic Studies; Science; Social Sciences; Life Sciences; Business Administration; Architecture; Engineering and Petroleum; Public Health; Medicine; Pharmacy; Dentistry; and Allied Health Sciences. A total of 744 undergraduate students, enrolled in these colleges, who were at least 18 years old, were chosen using convenience non-probability sampling. Considering non-responders and accounting for the sampling method, the sample was maximized to increase the external validity and generalizability (13). Postgraduate students were excluded to avoid potential bias due to differing characteristics compared to undergraduates. Recruitment was conducted from February to April 2023. The study was approved by the Health Sciences Center ethics committee at KU and Ministry of Health (MOH) in 9th of January 2025. Because the survey was distributed online via convenience sampling, the total number of students reached could not be tracked, and therefore a response rate was not calculable.

### 2.2 Variable measures and data collection

MIFCS was defined as Botox injections (for wrinkle smoothing and brow lifts), facial filler injections (for lip, chin, jawline, under-eye, cheek, and nose shaping), facelifts (using filler injections, i.e., “liquid facelift”), and permanent makeup (such as cosmetic and eyebrow tattooing). Other minimally invasive procedures such as chemical peels, microneedling, laser/light therapy, and thread lifts were not included, as they are less commonly reported among young adults and were beyond the primary scope of this study. An online questionnaire assessed undergoing MIFCS, which measured demographic characteristics, physical and mental health history, and evaluated the participant’s mental health status (depression, anxiety, and self-esteem). Mental health was measured using the Depression, Anxiety, and Stress Scale-21 Items (DASS-21) (14), focusing on depression and anxiety but excluding stress. Self-esteem was measured using the Rosenberg Self-Esteem Scale (RSE) (15). These measures serve as validated screening tools but are not substitutes for clinical diagnoses. The online questionnaire, available in both Arabic and English, was distributed via a link on WhatsApp and through barcode scanning (QR code). Detailed descriptions of the questionnaire and variables are provided in Supplementary file 1 (A description of the study variables) and Supplementary file 2 (The questionnaire).

### 2.3 Statistical analysis

Eighteen variables were measured to assess how well they predicted undergoing MIFCS. Age was categorized into three categories: (18–19, 20–21, and 22 or older); College affiliation was classified into Arts, Sciences, and Health Sciences Center (HSC). After obtaining the scores for depression, anxiety, and self-esteem, they were recoded as binary variables: depression and anxiety were categorized into “Normal” and “Not Normal,” and self-esteem was categorized into “Normal or Above Normal” and “Not Normal.” MIFCS was tabulated with each predictor for descriptive analyses, producing frequencies and relative frequencies (percentages). In addition, the Chi-square test (*X*^2^) was used to calculate *p*-values between MIFCS and each predictor. Univariate simple logistic regression analyses assessed the association between undergoing MIFCS and each independent predictor. Predictors with statistical significance (*α* = 0.1) in the univariate analyses were incorporated into the multiple logistic regression analysis. Backward elimination was applied to obtain the simplest, parsimonious, best-fit model (*α* = 0.05). The likelihood ratio test was performed to evaluate the goodness of fit. The final model exhibited no issues with multicollinearity, and specification errors were checked to ensure correct predictor inclusion. Statistical analyses were conducted using STATA software developed by StataCorp LP, a company in College Station, Texas.

## 3 Results

### 3.1 Participants characteristics

A total of 744 undergraduates participated in the study, of whom (*n* = 153, 20.6%) reported undergoing at least one minimally invasive procedure, while (*n* = 591, 79.4%) had never undergone such procedures. The majority of the participants were female students (*n* = 634, 85.2%), Kuwaiti nationals (*n* = 648, 87.1%), between 18 to 19 years old, comprising 281 (37.8%) of the sample, while 291 (39.1%) studied arts (Table 1). The participants’ residences were distributed across the six governorates, with varying percentages ranging from 9.9% (*n* = 74) in Mubarak Alkabeer to 20.8% (*n* = 155) in Farwaniya. Most participants were single (*n* = 682, 91.7%) and perceived their family income as average (*n* = 543, 73%). The primary sources of monthly income were family and university wages (*n* = 426, 57.3%). Regarding family dynamics, over 40% of participants’ parents had attained a bachelor’s degree or diploma, 79.8% (*n* = 594) reported a good relationship with their parents, and 59.4% (*n* = 442) had not experienced bullying. Concerning health history, 89.2% (*n* = 664) had no physical disorders, 75.7% (*n* = 563) had no mental disorders, and 89.4% (*n* = 665) had no family history of mental disorders. Mental health assessments showed that 64.4% (*n* = 479) of participants reported elevated levels of depressive symptoms and 68.8% (*n* = 512) reported elevated levels of anxiety symptoms. Self-esteem was normal in 69.9% (*n* = 520) of participants.

**Table 1:** Participants’ characteristics and the association with undergoing minimally invasive facial cosmetic surgery (MIFCS).

Characteristic	Subgroup totals		MIFCS				*P*-value
			No		Yes		
	*n*	(%)	*n*	(%)	*n*	(%)	
MIFCS	744	(100.0)	591	(79.4)	153	(20.6)	
Age group (years)							<0.001*
18–19	281	(37.8)	258	(91.8)	23	(8.2)	
20–21	252	(33.9)	188	(74.6)	64	(25.4)	
≥ 22	211	(28.4)	145	(68.7)	66	(31.3)	
Gender							0.001*
Male	110	(14.8)	100	(90.9)	10	(9.1)	
Female	634	(85.2)	491	(77.4)	143	(22.6)	
Nationality							0.018*
Non-Kuwaiti	96	(12.9)	85	(88.5)	11	(11.5)	
Kuwaiti	648	(87.1)	506	(78.1)	142	(21.9)	
College categories							0.009*
Arts	291	(39.1)	217	(74.6)	74	(25.4)	
Sciences	278	(37.4)	223	(80.2)	55	(19.8)	
Hsc	175	(23.5)	151	(86.3)	24	(13.7)	
Governorate							0.440
Capital	150	(20.2)	127	(84.7)	23	(15.3)	
Ahmadi	134	(18.0)	106	(79.1)	28	(20.9)	
Farwaniya	155	(20.8)	124	(80.0)	31	(20.0)	
Hawalli	107	(14.4)	84	(78.5)	23	(21.5)	
Jahra	124	(16.7)	96	(77.4)	28	(22.6)	
Mubarak Alkabeer	74	(9.9)	54	(73.0)	20	(27.0)	
Marital status							<0.001*
Single	682	(91.7)	556	(81.5)	126	(18.5)	
Married	62	(8.3)	35	(56.4)	27	(43.6)	
Perception of family income							0.046*
Average	543	(73.0)	441	(81.2)	102	(18.8)	
Below average	38	(5.1)	25	(65.8)	13	(34.2)	
Above average	163	(21.9)	125	(76.7)	38	(23.3)	
Source of monthly income							0.043*
University wage	248	(33.3)	204	(82.3)	44	(17.7)	
Family and university wage	426	(57.3)	339	(79.6)	87	(20.4)	
Family only or job salary	70	(9.4)	48	(68.6)	22	(31.4)	
Father’s education							0.452
Middle school or lower	106	(14.2)	80	(75.5)	26	(24.5)	
High school	176	(23.7)	138	(78.4)	38	(21.6)	
Undergraduate/diploma	312	(41.9)	256	(82.0)	56	(18.0)	
Postgraduate	150	(20.2)	117	(78.0)	33	(22.0)	
Mother’s education							0.660
Middle school or lower	108	(14.5)	81	(75.0)	27	(25.0)	
High school	156	(21.0)	125	(80.1)	31	(19.9)	
Undergraduate/diploma	364	(48.9)	293	(80.5)	71	(19.5)	
Postgraduate	116	(15.6)	92	(79.3)	24	(20.7)	
Relationship with parents							0.039*
Good	594	(79.8)	481	(81.0)	113	(19.0)	
Average or bad	150	(20.2)	110	(73.3)	40	(26.7)	
Bullying							0.203
No	442	(59.4)	358	(81.0)	84	(19.0)	
Yes	302	(40.6)	233	(77.1)	69	(22.9)	
History of physical disorders							0.012*
No	664	(89.2)	536	(80.7)	128	(19.3)	
Yes	80	(10.8)	55	(68.8)	25	(31.2)	
History of mental disorders							0.424
No	563	(75.7)	451	(80.1)	112	(19.9)	
Yes	181	(24.3)	140	(77.3)	41	(22.7)	
Family history of mental disorders							0.010*
No	665	(89.4)	537	(80.8)	128	(19.2)	
Yes	79	(10.6)	54	(68.3)	25	(31.7)	
Depression							0.047*
Normal	265	(35.6)	221	(83.4)	44	(16.6)	
Not normal	479	(64.4)	370	(77.2)	109	(22.8)	
Anxiety							<0.001*
Normal	232	(31.2)	203	(87.5)	29	(12.5)	
Not normal	512	(68.8)	388	(75.8)	124	(24.2)	
Self-esteem							0.443
Normal	520	(69.9)	417	(80.2)	103	(19.8)	
Above normal	130	(17.5)	104	(80.0)	26	(20.0)	
Below normal	94	(12.6)	70	(74.5)	24	(25.5)	

*Significant *p*-values (*α* = 0.1) for categorical variables obtained from *X*^2^ test.

### 3.2 Prevalence of MIFCS procedures

**Figure 1:**
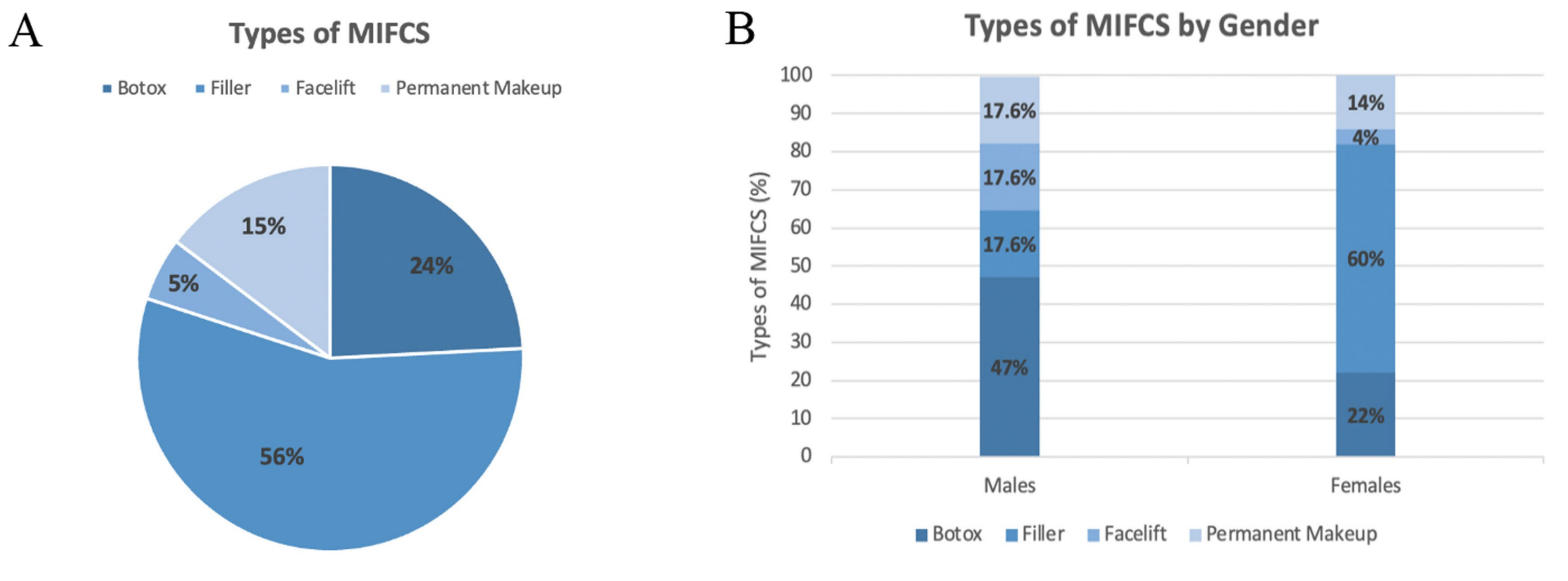
Percentages of minimally invasive facial cosmetic surgery (MIFCS) among Kuwait University undergraduates. **(A)** Pie chart illustrating the distribution of MIFCS procedures performed by students. **(B)** Bar chart of gender-specific distribution of MIFCS procedures performed by students. Percentages refer to the total number of procedures performed.

Of the total number of procedures performed, filler (*n* = 85/153, 55.8%) was the most common procedure undergone, followed by Botox (*n* = 37/153, 24.2%), permanent makeup (*n* = 22/153, 14.7%), and facelift (*n* = 8/153, 5.3%), which was the least frequent procedure performed (Figure 1A). Among females, filler was the most prevalent procedure performed (60%), while Botox was more common among males (47%) (Figure 1B). The distribution of other procedures (filler, facelift, permanent makeup) was evenly distributed among males; however, among females, facelifts were less frequent compared to other MIFCS procedures (4% vs. 14% permanent makeup, 22% Botox, and 60% filler).

**Figure 2:**
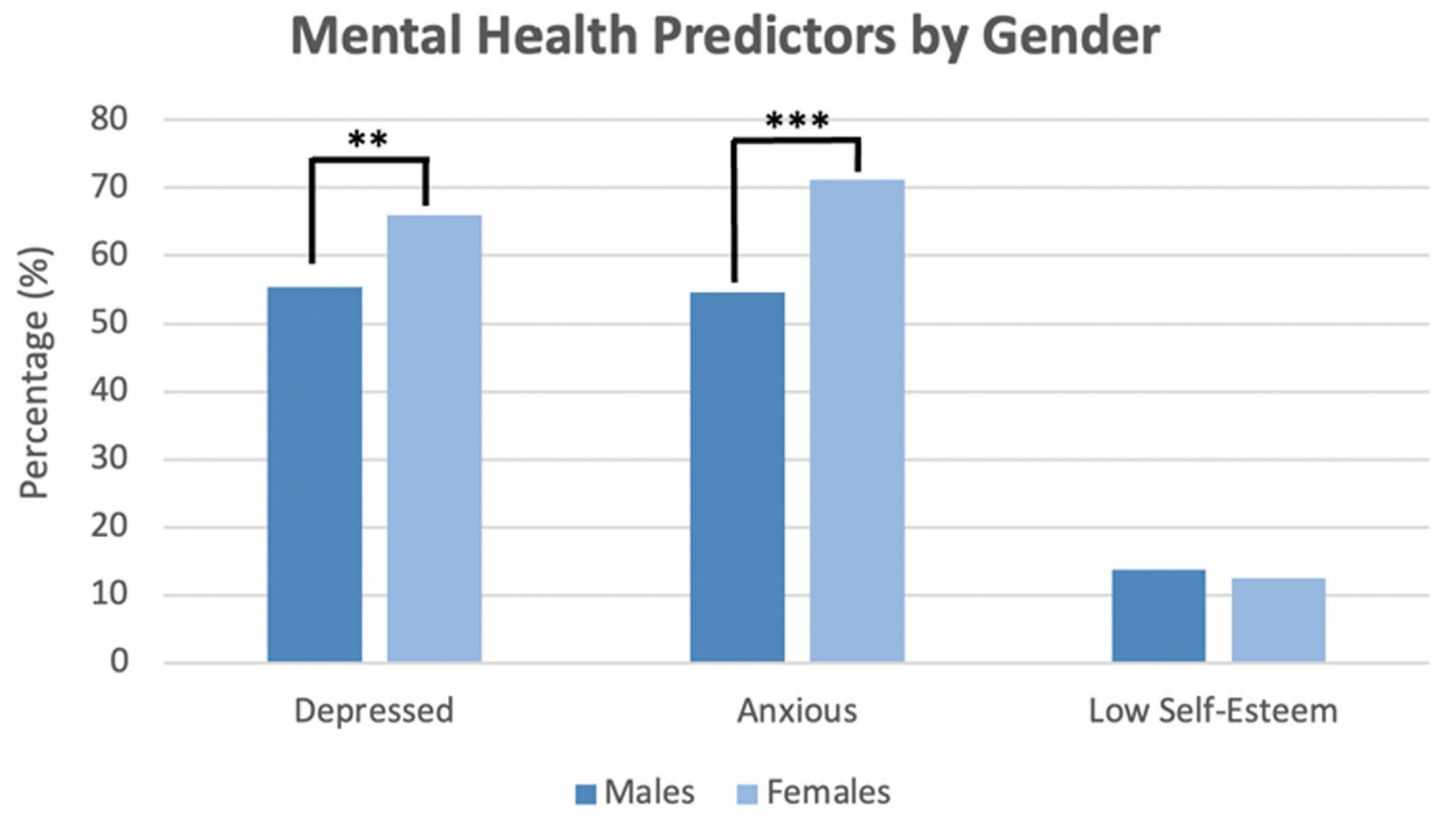
Gender-specific levels of depressive symptoms, anxiety symptoms, and self-esteem of students. *p*-values assessing the association between variables and gender were obtained by *X*^2^***p < 0.01, **p < 0.01.

Figure 2 shows that females had a significantly higher levels of depression and anxiety than males: depression (65.9% vs. 55.5%) and anxiety (71.3% vs. 54.6%), respectively. No statistically significant difference was found between females and males regarding low self-esteem (12.5% vs. 13.6%).

### 3.3 Factors associated with MIFCS

Variables significantly associated with undergoing MIFCS include individuals aged 20–21 years having higher odds than those aged 18–19 years (aOR 4.2; 95% CI 2.47–7.16), and those aged 22 years and above having 5.2 times higher odds (aOR 5.2; 95% CI 2.98–8.96) while adjusting for the effect of other variables (Table 2). Females were four times more likely than males to have MIFCS (aOR 4.0, 95% CI 1.95–8.24). Students studying in one of the colleges of the HSC had a 56% lower chance of undergoing MIFCS than those in Arts (aOR 0.44; 95% CI 0.25–0.76). Being married increased the odds of undergoing MIFCS compared to being single (aOR 2.4; 95% CI 1.32–4.29). Participants with a history of physical conditions were more likely to undergo MIFCS (aOR 1.8; 95% CI 1.00–1.11), and those with a family history of mental disorders (aOR 1.87; 95% CI 1.06–3.31). Participants with anxiety were 2.3 times more likely to undertake MIFCS than non-anxious participants (aOR 2.3; 95% CI 1.42–3.64).

**Table 2:** Multivariate regression analysis: factors associated with adjusted odds of undergoing minimally invasive facial cosmetic surgery (MIFCS).

Characteristics	Odds ratio	95% Confidence interval	*p*-value
Age group (years)			
<20	[Reference]		
20–21	4.20	(2.47, 7.16)	<0.001*
≥ 22	5.17	(2.98, 8.96)	<0.001*
Gender			
Male	[Reference]		
Female	4.01	(1.95, 8.24)	<0.001*
College categories			
Arts	[Reference]		
Sciences	0.81	(0.53, 1.25)	0.352
Hsc	0.44	(0.25, 0.76)	0.003*
Marital status			
Single	[Reference]		
Married	2.38	(1.32, 4.29)	0.004*
History of physical disorders			
No	[Reference]		
Yes	1.77	(1.00, 1.11)	0.049*
Family history of mental disorders			
No	[Reference]		
Yes	1.87	(1.06, 3.31)	0.031*
Anxiety			
Normal	[Reference]		
Not normal	2.27	(1.42, 3.64)	0.001*

*Significant *p*-values (*α* = 0.05).

## 4 Discussion

### 4.1 Key findings

To date, we believe this study to be the first to explore the relationship between MIFCS and psychological health in college students in Kuwait, using validated measures to assess levels of depression, anxiety, and self-esteem. These factors are critical in understanding students’ motivations for undergoing cosmetic procedures, with significant implications for healthcare professionals to address mental health concerns alongside the rising trend of cosmetic surgery.

### 4.2 Comparison with previous studies and implication for practice and policy

Our study found a prevalence of 20.6% among KU undergraduates, notably higher than the 7% reported in the Al-Rifaai study (12). This difference could be due to the small sample size in the Al-Rifaai study. In comparison, the prevalence of MIFCS among students in China was only 3%, possibly because of differences in female-to-male sex ratios (6:1 in our study, 1:1 in the Chinese study) (4). Future research should seek a more balanced sample to examine these trends more closely. This study reported filler and Botox injections as the most prevalent procedures undertaken by participants who had MIFCS procedures. Population-based prevalence estimates are scarce; thus, data obtained from the International Society of Aesthetic Plastic Surgery (ISAPS) could aid in understanding the global trend of cosmetic procedures. Consistent with our findings and international trends, Botox injections are the most frequently performed non-surgical procedure worldwide (8.88 million procedures in 2023), followed by filler injections (5.56 million procedures in 2023); this ranking also characterizes Middle Eastern countries included in the ISAPS dataset (16). This pattern holds across sexes and age groups, indicating broad global uptake.

The study sample included participants from only one ethnic background limiting the possibility to compare MIFCS procedures between ethnicities. The ISAPS survey shows that the United States (US), Brazil, and South Korea have the highest numbers of aesthetic procedures worldwide (16). Data by ethnicity are limited; in the US, most procedures have been reported among White/Caucasian patients, with growing uptake among Asian, Hispanic, and Black groups (17). In the Middle East, demand is also rising, but population-level data are lacking (16). Therefore, Arabs are not currently among the global per-capita leaders, and ethnic comparisons should be interpreted with caution. More population-based studies are needed to understand ethnic and regional differences in minimally invasive procedure prevalence.

The prevalence of depression and anxiety in our study was 64 and 69%, respectively. This surpasses results reported in similar studies in Saudi Arabia (36 and 41.5%) (18) and the UAE (38 and 55%) (19), respectively. With regards to self-esteem, a study in the UAE observed that 17% of students had low self-esteem (20), which was relatively similar (13%) to that reported in this study. However, the UAE study reported that 10% of students had high self-esteem (above normal), almost two times lower than our finding (18%). This could result from the bidirectional relationship between self-esteem and cosmetic procedures, in which cosmetic surgeries could enhance self-esteem (21). As mental health issues continue to affect a large proportion of college students, our study highlights the importance of increasing access to mental health counseling services on campuses to support students’ well-being.

Our results indicated that students at the HSC—including those studying medicine, dentistry, pharmacy, public health, or allied health—showed a 56% lower tendency to undergo MIFCS compared to students at the arts colleges, highlighting the potential influence of academic discipline on students’ attitudes toward cosmetic procedures. Similarly, a study in Norway found that law students were more likely to partake in aesthetic procedures in comparison with medical students (22). Another study suggested that medical students were more likely to have negative attitudes toward cosmetic surgery and were less likely to consider it for themselves (23).

The current study saw a fourfold increase in MIFCS among females as compared to males. College students’ attitudes regarding cosmetic procedures can be explained by the fact that women are more pressured by society to maintain particular standards of beauty than men (24, 25). In addition, Rita Davai and colleagues, who studied the impact of cosmetic surgery on married women in Iran found that marriage satisfaction was positively impacted by aesthetic surgery (26). This could be the rationale behind the higher rate of cosmetic surgery among married women.

Regarding the association between age and MIFCS, students aged 20 and above were more likely to undergo cosmetic procedures compared to students aged 18 and 19. This finding aligns with a French study that demonstrated increasing concerns about facial aging as women age (27). To combat the growing trend of cosmetic procedures, public health campaigns should prioritize addressing age-related concerns, promoting positive body image among young adults, encouraging informed decision-making, ensuring patient safety, and providing support for those who choose to undergo cosmetic procedures.

Our results indicated a significant association between a history of physical disorders, as well as a family history of mental disorders, and undergoing MIFCS. This contrasts with findings from a Chinese study, which found no link between the two factors and MIFCS (4). There is a noticeable gap in research concerning the relationship between the history of physical disorders and undergoing MIFCS. One possible explanation for the association with a family history of mental disorders is that individuals with a family history of mental disorders may be more likely to experience body image issues, low self-esteem, and dissatisfaction with their appearance, leading them to seek cosmetic surgery to improve their self-confidence. It is crucial to note that while cosmetic surgery may provide temporary relief from body image concerns, it does not address the underlying psychological issues contributing to an individual’s dissatisfaction with their appearance. Therefore, individuals with a family history of mental disorders considering cosmetic surgery should seek support and treatment for any underlying mental health issues.

An important factor not assessed in our study is BDD, which is significantly more prevalent among cosmetic surgery patients (up to 15–20%) than in the general population (~2%) (11). Unlike general depression or anxiety, BDD is closely tied to body image distortion and often persists or worsens after cosmetic procedures, leading to ongoing dissatisfaction (10). Our findings of elevated levels of depression and anxiety may partly reflect unrecognized BDD, underscoring the importance of screening for this condition.

### 4.3 Limitations

While this study provides valuable insight into the field, several limitations must be addressed. First, the cross-sectional design precludes causal inferences, despite indicating a correlation between MIFCS and psychological predictors. Longitudinal research is required to investigate these correlations further. Second, data were obtained solely from Kuwait University colleges, which limits their applicability to students at other universities, particularly private colleges. Third, the snowball sampling approach utilized may not have resulted in a representative sample, with males underrepresented. Another limitation is the overrepresentation of females (85%), which is higher than their proportion in the general student body. This may introduce response bias and limit the representativeness of the findings. This study used validated measures, however, no assessment of BDD was included. As BDD is known to strongly influence both the decision to undergo cosmetic procedures and postoperative outcomes, this omission represents a limitation of the present study. Additionally, the study sample consisted of participants from a single ethnic group, which may limit the generalizability of the findings. Cultural perceptions and attitudes toward cosmetic procedures can vary across populations, and therefore, the results may not be directly applicable to other ethnic groups. Future studies including more diverse populations are needed to confirm and extend these findings.

## 5 Conclusion

The observed multifactorial nature and higher prevalence of MIFCS in Kuwait point to the need for a deeper comprehension of the psychological factors influencing cosmetic procedures. Key predictors include being female, older, married, and having personal or family histories of physical and mental health issues, particularly anxiety. Interestingly, students in medical academic fields had lower odds of undergoing MIFCS, suggesting a protective effect of medical education. Understanding the psychological variables that affect cosmetic procedures is vital for effective counseling and therapeutic practice, ensuring comprehensive care for patients’ mental well-being.

Future research should explore additional factors, including the influence of social media—shown to affect young women’s desire for cosmetic surgery (28) as well as body dysmorphic disorder. Researchers should look at the association between mental health and MIFCS in vulnerable groups, such as individuals with pre-existing mental health disorders or traumatic life events. Comparative studies of MIFCS outcomes with other types of cosmetic procedures or surgeries are recommended as well.

## Data Availability

The raw data supporting the conclusions of this article will be made available by the authors, without undue reservation.

## References

[1] Zhong Y Liang F. Analysis of cosmetic effect of botulinum toxin type a masseter fixed point injection combined with hyaluronic acid mentalfilling injection for remodeling female maxillofacial contour. Womens Health Res. (2020) 13:112–3.

[2] Pearlman RL Wilkerson AH Cobb EK Morrissette S Lawson FG Mockbee CS. Factors associated with likelihood to undergo cosmetic surgical procedures among young adults in the United States: a narrative review. Clin Cosmet Investig Dermatol. (2022) 15:859–77. doi: 10.2147/CCID.S358573, PMID: 35592730 PMC9112174

[3] AlShamlan NA AlOmar RS Al-Sahow AZ AlShamlan AA Almir BM Al-Johani WM. Cosmetic surgeries and procedures among youth in Saudi Arabia: a cross-sectional study of undergraduate university students in the Eastern Province. Postgrad Med J. (2022) 98:434–40. doi: 10.1136/postgradmedj-2020-139618, PMID: 33541935

[4] Jin X Twayigira M Zhang W Gao X Luo X Xu H. Prevalence and associated factors of minimally invasive facial cosmetic surgery in Chinese college students. BMC Psychiatry. (2022) 22:1–10. doi: 10.1186/s12888-021-03676-3, PMID: 35012505 PMC8750801

[5] Auer D. Understanding body image from a psychosocial stance: how this connects with patients requesting cosmetic procedures. J Aesthetic Nursing. (2020) 9:128–31. doi: 10.12968/joan.2020.9.3.128

[6] Cooley CH. Human nature and the social order. UK (Milton Park, Abingdon, Oxfordshire): Routledge (2017).

[7] DeYoung PA. Understanding and treating chronic shame: A relational/neurobiological approach. New York: Routledge (2015).

[8] Lee K Guy A Dale J Wolke D. Adolescent desire for cosmetic surgery: associations with bullying and psychological functioning. Plast Reconstr Surg. (2017) 139:1109–18. doi: 10.1097/PRS.0000000000003252, PMID: 28445361

[9] Sharp G Tiggemann M Mattiske J. The role of media and peer influences in Australian women's attitudes towards cosmetic surgery. Body Image. (2014) 11:482–7. doi: 10.1016/j.bodyim.2014.07.009, PMID: 25129686

[10] Rehman U Perwaiz I Sarwar MS Brennan PA. Mental health screening in facial cosmetic surgery: a narrative review of the literature. Br J Oral Maxillofac Surg. (2023) 61:455–63. doi: 10.1016/j.bjoms.2023.05.003, PMID: 37442708

[11] Veale D Gledhill LJ Christodoulou P Hodsoll J. Body dysmorphic disorder in different settings: a systematic review and estimated weighted prevalence. Body Image. (2016) 18:168–86. doi: 10.1016/j.bodyim.2016.07.003, PMID: 27498379

[12] Al-Rifaai J Alfowzan NA. Attitudes and awareness towards cosmetic surgery among college students in Kuwait. Eur J Biology Med Sci Res. (2022) 10:22–32. doi: 10.37745/ejbmsr.2013/vol10n42232

[13] Cavana R Delahaye B Sekeran U. Applied business research: Qualitative and quantitative methods. Australia: John Wiley & Sons (2001).

[14] Lovibond SH. Manual for the depression anxiety stress scales Sydney psychology foundation. Sydney, Australia (1995).

[15] Rosenberg M. Society and the adolescent self-image. Princeton, NJ: Princeton University Press (1965).

[16] ISAPS. ISAPS international survey on aesthetic/cosmetic procedures performed in 2023: International Society of Aesthetic Plastic Surgery; (2023). Available online at: https://www.isaps.org/media/rxnfqibn/isaps-global-survey_2023.pdf.

[17] American Society of Plastic Surgeons (2021). Procedures by Ethnicity: Cosmetic Procedures Performed in 2020. Available at: https://www.plasticsurgery.org/documents/News/Statistics/2020/cosmetic-procedures-ethnicity-2020.pdf

[18] Hakim RF Alrahmani DA Ahmed DM Alharthi NA Fida AR Al-Raddadi RM. Association of body dysmorphic disorder with anxiety, depression, and stress among university students. J Taibah Univ Med Sci. (2021) 16:689–94. doi: 10.1016/j.jtumed.2021.05.008, PMID: 34690648 PMC8498710

[19] Al Marzouqi AM Otim ME Alblooshi A Al Marzooqi S Talal M Wassim F. State of emotional health disorders of undergraduate students in the United Arab Emirates: a cross-sectional survey. Psychol Res Behav Manag. (2022) 15:1423–33. doi: 10.2147/PRBM.S365012, PMID: 35698566 PMC9188331

[20] Vally Z. Generalized problematic internet use, depression, and explicit self-esteem: evidence from the United Arab Emirates. Neurol Psychiatry Brain Res. (2019) 33:93–100. doi: 10.1016/j.npbr.2019.07.002

[21] Kazeminia M Salari N Heydari M Akbari H Mohammadi M. The effect of cosmetic surgery on self-esteem and body image: a systematic review and meta-analysis of clinical trial studies. Eur J Plast Surg. (2023) 46:25–33. doi: 10.1007/s00238-022-01987-6

[22] Almeland SK Guttormsen AB de Weerd L Nordgaard HB Freccero C Hansson E. Plastic surgery in the Norwegian undergraduate medical curriculum: students’ knowledge and attitudes. A nationwide case-control study. J Plast Surg Hand Surg. (2017) 51:136–42. doi: 10.1080/2000656X.2016.1203330, PMID: 27387588

[23] Jabaiti S Hamdan-Mansour AM Isleem UN Altarawneh S Araggad L Al Ibraheem GA. Impact of plastic surgery medical training on medical students’ knowledge, attitudes, preferences, and perceived benefits: comparative study. J Public Health Res. (2021) 10:1927. doi: 10.4081/jphr.2021.1927 33759481 PMC8314676

[24] De Vries DA Peter J. Women on display: the effect of portraying the self online on women’s self-objectification. Comput Hum Behav. (2013) 29:1483–9. doi: 10.1016/j.chb.2013.01.015

[25] Gervais SJ Vescio TK Förster J Maass A Suitner C. Seeing women as objects: the sexual body part recognition bias. Eur J Soc Psychol. (2012) 42:743–53. doi: 10.1002/ejsp.1890

[26] Davai NR Ganji K Kalantar-Hormozi A Abbaszadeh-Kasbi A. The impact of cosmetic surgery on married women’s marital satisfaction and self-concept. World J Plast Surg. (2018) 7:171. doi: 10.29252/wjps.7.3.337 30083499 PMC6066701

[27] Ehlinger-Martin A Cohen-Letessier A Taïeb M Azoulay E du Crest D. Women's attitudes to beauty, aging, and the place of cosmetic procedures: insights from the QUEST observatory. J Cosmet Dermatol. (2016) 15:89–94. doi: 10.1111/jocd.12192, PMID: 26566891

[28] Walker CE Krumhuber EG Dayan S Furnham A. Effects of social media use on desire for cosmetic surgery among young women. Curr Psychol. (2021) 40:3355–64. doi: 10.1007/s12144-019-00282-1

