# Supplementary material for: Prevalence of minimally invasive facial cosmetic surgery and its association with mental health among college students in Kuwait
Source: Front Public Health. 2025 Oct 9;13:1678308. doi: 10.3389/fpubh.2025.1678308 (PMC12545008; doi:10.3389/fpubh.2025.1678308)
Supplement: Supplementary file 1 [file Supplementary_file_1.DOCX]

# Supplementary file 1: A description of the study variables

The questionnaire consisted of four sections. Section A: Demographic characteristics; Section B: Physical and mental health history; Section C: Psychological Health-Depression, Anxiety, and Self-esteem; and Section D: Undergoing MIFCS.

Demographic factors included age (years), gender (male/female), nationality (Kuwaiti/Non-Kuwaiti), marital status (single/married/divorced/widowed), governorate (AlAhmadi, Capital, Farwaniya, Hawalli, Jahra, Mubarak AlKabeer), college (College of Arts/ Education/ Law/ Sharia and Islamic Studies/ Science/ Social Sciences/ Life Sciences/ Business Administration/ Architecture/ Engineering and Petroleum/ Public Health/ Medicine/ Pharmacy/ Dentistry/ Allied Health Sciences), perception of family's monthly income (below average/ average/ above average), personal monthly income source (allowance from family only/ student university wage only/ allowance from family and student university wage/ job salary), parents' educational level (middle school/ high school/undergraduate degree or diploma/postgraduate degree), perception of relationship with parents (good/average/bad), experienced bullying regarding physical features (no/yes).

Health history factors included the history of physical disorders (no/yes), history of mental disorders (no/yes), and family history of mental disorders (no/yes).

Information about participants' psychological health, including depressive and anxiety symptoms, was gathered using the self-report Depression, Anxiety, and Stress Scale – 21 Items (DASS-21). The individual had to specify if a symptom had been present throughout the last week when filling out the DASS. The Depression, Anxiety, and Stress Scale - 21 Items (DASS-21) is a collection of three self-report tests aimed at evaluating depression, anxiety, and stress; however, this assessment does not replace a clinical diagnosis. The rating scale is coded as follows: 0 never, 1 sometimes, 2 often, and 3 almost always. For each response, scores will be added up, and then multiplied by 2 to obtain the final score. For depression, scores below 9 indicate normal status, 10-13 indicate mild depression, 14-20 moderate depression, 21-27 severe, and 28 and above indicate severely extreme depression, whereas for anxiety, scores below 7 indicate normal status, 8-9 mild anxiety, 10-14 moderate anxiety, 15-19 severe anxiety, and 20 and above indicate extremely severe anxiety. The Rosenberg Self-Esteem Scale (RSE), a 10-item scale that assesses both positive and unfavorable thoughts about oneself to determine overall self-worth, was used to measure the participants' self-esteem. Each question has a 4-point Likert scale response option, ranging from strongly agree to strongly disagree. Items 2, 5, 6, 8, and 9 are reversed scored, meaning that for items 1,3,4,7, and 10, scores are calculated as follows: Strongly agree = 3, Agree = 2, Disagree = 1, Strongly disagree = 0, whereas for items (which are reversed in valence): Strongly agree = 0, Agree = 1, Disagree = 2, Strongly disagree = 3. Higher scores indicate higher self-esteem. The scale has a range of 0 to 30. Scores below 15 indicate low self-esteem; values between 15 and 25 are considered normal.

Information about whether or not participants had undergone cosmetic surgery was obtained from a question with (no/yes) options, and the type of surgery was obtained from a question with a checklist of almost all possible minimally invasive facial cosmetic surgeries we think are popular in Kuwait; options included Botox injections such as wrinkle smoothing and brow lift; facial filler injections such as lip, chin, jawline, under-eye, cheek, and nose-shaping fillers; face-lift, eyebrow tattooing, and permanent make-up.
