# Supplementary material for: Prevalence of minimally invasive facial cosmetic surgery and its association with mental health among college students in Kuwait
Source: Front Public Health. 2025 Oct 9;13:1678308. doi: 10.3389/fpubh.2025.1678308 (PMC12545008; doi:10.3389/fpubh.2025.1678308)
Supplement: Supplementary file 2 [file Supplementary_file_2.DOCX]

**Supplementary file 2: Questionnaire:**

**Prevalence of Minimally Invasive Facial Cosmetic Surgery (MIFCS) and its Association with Mental Health among College Students: A Cross-Sectional Study**

**Questionnaire**

Dear participant,

Thank you for your interest in taking part in this online survey.

This is an informed consent form that discusses the study’s aims and procedures. By signing this consent, you are consenting to take part in our study. You are being invited to take part in a study about minimally invasive facial cosmetic surgery and your mental and psychological health. This study will involve answering a short 7–10-minute online questionnaire. It is the first study in Kuwait that will assess the association between mental health and undergoing cosmetic facial surgery among Kuwait University undergraduates.

There are no risks to you if you participate in this research. Your participation will increase knowledge about this important issue. All information collected will remain confidential. Neither your name nor address will be recorded in any assessment. There is no obligation or compulsion for you to participate, and you have the freedom to agree or not agree to participate. This will not have any effect on your academic standing. You may withdraw from the research at any time. This research does not include any medical experiments, taking biological samples, or intervening in any treatment plan set by your physician.

Your decision to participate in this study is completely voluntary and you have the right to end your participation at any time. Your participation in this study will be reported cumulatively.

By selecting the box, you agree and confirm that:

- You are at least 18 years of age
- You are an undergraduate student at Kuwait University

Please indicate ( ✓ ) below if you wish to participate or decline to do so:

I wish to participate. Name of participant:

I do not wish to participate. Signature of participant:

Thank You for Your Cooperation . . .

Student name(s): Aisha Saadallah, Dina Ibrahim, Zainab Awada

Supervised by: Dr. Eiman Al-Awadhi Date:

| **Section A:** Socio-demographic characteristics |
| --- |

| 1. What is your age -------------------- ? |
| --- |

| 1. What is your gender? | |
| --- | --- |
| - Male | - Female |

| 1. What is your nationality? | |
| --- | --- |
| - Non-Kuwaiti | - Kuwaiti |

| 1. In which college do you study? | | |
| --- | --- | --- |
| - Arts | - Education | - Law |
| - Islamic Studies | - Sciences | - Social Sciences |
| - Life Sciences | - Business Administration | - Architecture |
| - Engineering | - Public Health | - Medicine |
| - Pharmacy | - Dentistry | - Allied Health Sciences |

| 1. In which governorate do you live? | | |
| --- | --- | --- |
| - Al-Ahmadi | - Capital | - Farwaniya |
| - Hawalli | - Jahra | - Mubarak Al-Kabeer |

| 1. What is your marital status? | |
| --- | --- |
| - Single | - Married |
| - Divorced | - Widowed |

| 1. What do you perceive your monthly family income to be? | | |
| --- | --- | --- |
| - Below Average | - Average | - Above Average |

| 1. What is the source of your monthly income? | |
| --- | --- |
| - Family Allowance | - Student university wage only |
| - Family allowance and university wage | - Job salary |

| 1. What is your father’s educational level? | |
| --- | --- |
| - Middle school or lower | - High school |
| - Undergraduate degree/diploma | - Postgraduate degree |

| 1. What is your mother’s educational level? | |
| --- | --- |
| - Middle school or lower | - High school |
| - Undergraduate degree/diploma | - Postgraduate degree |

| 1. How do you perceive your relationship with your parents to be? | | |
| --- | --- | --- |
| - Good | - Average | - Bad |

| 1. Have you experienced any type of bullying regarding your physical features? | |
| --- | --- |
| - No | - Yes |

| **Section B:** Physical and Mental Health History |
| --- |

| 1. Do you have a history of physical disorders (e.g., diabetes, hypertension, heart disease, cancer, epilepsy, Tourette syndrome, etc.)? | |
| --- | --- |
| - No | - Yes |

| 1. Do you have a history of mental disorders (e.g., depression, anxiety, bipolar disorder, schizophrenia, post-traumatic stress disorder, eating disorders, etc.)? | |
| --- | --- |
| - No | - Yes |

| 1. Do you have a family history of mental disorders? | |
| --- | --- |
| - No | - Yes |

| **Section C:** Psychological Health |
| --- |

| **Instructions:** Please read each statement and select the phrase (never, sometimes, often, almost always) to indicate how much the statement applied to you over the past week. |
| --- |

|  | **Never** | **Sometimes** | **Often** | **Always** |
| --- | --- | --- | --- | --- |
| 1. I couldn’t seem to experience any positive feeling at all |  |  |  |  |
| 1. I found it difficult to work up the initiative to do things |  |  |  |  |
| 1. I felt that I had nothing to look forward to |  |  |  |  |
| 1. I felt down-hearted and blue |  |  |  |  |
| 1. I was unable to become enthusiastic about anything |  |  |  |  |
| 1. I felt I wasn’t worth much as a person |  |  |  |  |
| 1. I felt that life was meaningless |  |  |  |  |
| 1. I was aware of dryness of my mouth |  |  |  |  |
| 1. I experienced breathing difficulty (e.g., excessively rapid breathing, breathlessness in the absence of physical exertion) |  |  |  |  |
| 1. I experienced trembling (eg, in the hands) |  |  |  |  |
| 1. I was worried about situations in which I might panic and make a fool of myself |  |  |  |  |
| 1. I felt I was close to panic |  |  |  |  |
| 1. I was aware of the action of my heart in the absence of physicalexertion (eg, sense of heart rate increase, heart missing a beat) |  |  |  |  |
| 1. I felt scared without any good reason |  |  |  |  |

| **Instructions:** Please select the appropriate answer for each statement, depending on whether you Strongly agree, agree, disagree, or strongly disagree with it. |
| --- |

|  | **Strongly Agree** | **Agree** | **Disagree** | **Strongly Disagree** |
| --- | --- | --- | --- | --- |
| 1. On the whole, I am satisfied with myself |  |  |  |  |
| 1. At times I think I am no good at all |  |  |  |  |
| 1. I feel that I have a number of good qualities |  |  |  |  |
| 1. I am able to do things as well as most other people |  |  |  |  |
| 1. I feel 1do not have much to be proud of |  |  |  |  |
| 1. I certainly feel useless at times |  |  |  |  |
| 1. I feel that I'm a person of worth |  |  |  |  |
| 1. I wish I could have more respect for myself |  |  |  |  |
| 1. All in all, I am inclined to think that I am a failure |  |  |  |  |
| 1. I take a positive attitude toward myself |  |  |  |  |

| Section D: Undergoing Minimally Invasive Facial Cosmetic Surgery |
| --- |

| 1. Have you ever undergone minimally invasive facial cosmetic surgery [Botox (wrinkle smoothing/brow lift), Filler (lip/chin/jawline/under-eye/cheek/nose-shaping), Facelift, Eyebrow tattoo, Permanent make up, others]? | |
| --- | --- |
| - No | - Yes |

| 1. If yes, what type of minimally invasive facial cosmetic surgery? | | |
| --- | --- | --- |
| - Botox (wrinkle smoothing/brow lift) | - Filler (lip/chin/jawline/under-eye/cheek/nose-shaping) | - Facelift |
| - Eyebrow tattoo | - Permanent make up | - Others, please specify |
